# CHILDHOOD HOUSING AND OLD AGE HEALTH AMONG CHINESE: THE MEDIATION ROLE OF ADULTHOOD LIFE HISTORY

**DOI:** 10.1093/geroni/igae098.2788

**Published:** 2024-12-31

**Authors:** Dexia Kong, Peiyi Lu, Vivian Weiqun Lou

**Affiliations:** The Chinese University of Hong Kong, Hong Kong, China (People’s Republic); The University of Hong Kong, Hong Kong, China (People’s Republic); The University of Hong Kong, Hong Kong, China (People’s Republic)

## Abstract

Housing insecurity is linked to worse health. However, very few studies examined this relationship from a lifecourse perspective and its underlying mechanisms. This study examined the association of childhood housing with old age health among Chinese and whether adulthood socioeconomic and medical history mediated that relationship. Respondents were middle-aged and older adults (aged 45+) from the China Health and Retirement Longitudinal Study (N=12,842). They were asked about their childhood housing conditions (e.g., if their houses had clean water, water closet, and electricity). Adulthood socioeconomic and medical history and middle- and old-age health were measured. Causal mediation analysis examined the pathways via which childhood housing affects health in later life. Results show about 28%-37% of respondents had access to electricity or energy for cooking/heating during their childhood. However, very few had access to clean water (6%) or water toilet (2%). Childhood better housing was directly associated with fewer depressive symptoms and better cognition in middle- and older age, and indirectly through increasing the likelihood of obtaining more education during adulthood. However, the proportion-mediated estimate had very wide confidence intervals to be certain. Other adulthood life history pathways (i.e., marital status, work and medical history) were not statistically significant. Our findings suggested targeting services among those who grew up in poor housing conditions may be crucial to promoting healthy aging.

